# New HIV peptide-based immunoassay resolves vaccine-induced seropositivity in HIV vaccine (Phase III) trial participants

**DOI:** 10.1186/1742-4690-9-S2-P120

**Published:** 2012-09-13

**Authors:** O Penezina, D Clapham, J Collins, V Kovalenko, N Krueger, IR Rodriguez-Chavez, MP Busch, AE Levin

**Affiliations:** 1 Immunetics, Boston, MA, USA; 2 NIH/NIDCR, Bethesda, MD, USA; 3 Blood Systems Research Institute, San Francisco, CA, USA

## Background

HIV Vaccine trials bring the significant risk of vaccine-induced HIV seropositivity(VISP) resulting in negative personal consequences for vaccinees. The overall rate of VISP in licensed EIA tests is reported as 41.7%(JAMA 2010;304:275-283). We have developed and modified the peptide-based HIV Selectest immunoassay(J.Virol 2006;80:2092-2099), which discriminates VISP from true HIV infection, in a format suitable for routine laboratory use, and have evaluated its performance on samples from three Phase III HIV vaccine trials.

## Methods

The HIV Selectest incorporated five synthetic peptides in a single well microplate ELISA. Serum panels evaluated comprised well-characterized HIV-positive sera from clades A,B and C, worldwide panels comprising all major clades, blood donor controls, and sera from vaccine and placebo recipients in RV144, Vax003 and Vax004 trials.

## Results

360 serum samples from the RV144 vaccine trial, including 170 samples from vaccinated subjects at the peak immune response, 120 pre-immune samples, and 70 subjects from the placebo group were tested on the HIV Selectest. One (1) subject(0.6%) among the vaccine recipient group yielded false-positive results, while 3 placebo recipients (4.3%) and 1 pre-immune serum sample (0.8%) were also false positive in the HIV Selectest. All false-positive samples demonstrated broad non-specific cross-reactivity that was not restricted to a particular HIV-specific peptide.

Similar results were obtained with samples from the VAX003 and VAX004 vaccine trials. One subject out of 87(1.2%) tested after the final vaccination(7^th^visit) at the peak of the immune response was detected as false positive. Two additional samples out of 96(2.1%) taken after the 4^th^visit were likewise detected as false-positive, bringing the average false-positive rate for both groups to 1.6%.

Blood donors yielded a statistically equivalent false-positive rate of 1.2%. Detection sensitivity for HIV positive samples was 96% among 648 serum samples representing different clades.

## Conclusion

The HIV Selectest ELISA has demonstrated significantly better discrimination of VISP than currently licensed HIV serologic assays.

